# Adverse childhood experiences (ACEs) are associated with forced and very early sexual initiation among Black women accessing publicly funded STD clinics in Baltimore, MD

**DOI:** 10.1371/journal.pone.0216279

**Published:** 2019-05-07

**Authors:** Kiyomi Tsuyuki, Noor A. Al-Alusi, Jacquelyn C. Campbell, DeMarjion Murry, Andrea N. Cimino, Argentina E. Servin, Jamila K. Stockman

**Affiliations:** 1 Division of Infectious Diseases and Global Public Health, School of Medicine, University of California, San Diego, La Jolla, California, United States of America; 2 School of Nursing, Johns Hopkins University, Baltimore, Maryland, United States of America; 3 Philander Smith College, Little Rock, Arkansas, United States of America; Stellenbosch University, SOUTH AFRICA

## Abstract

**Purpose:**

To examine the association between adverse childhood experiences (ACEs) and early sexual initiation.

**Methods:**

We analyzed retrospective data of (n = 241) Black women recruited from public STD clinics in Baltimore, MD. Multinomial logistic and linear regression models estimated associations between ACEs and early sexual initiation; contextual variables at initiation were examined as mediators.

**Results:**

Twelve percent of our sample reported very early sexual initiation (11–12 years) and 29% reported early sexual initiation (13–14 years). Each additional ACE reported was associated with greater risk of very early sexual initiation (RRR = 1.49; 95%CI:1.23,1.80). Specifically, emotional abuse (RRR = 3.71; 95%CI:1.55,8.89), physical abuse (RRR = 9.45; 95%CI:3.56,25.12), sexual abuse (RRR = 8.60; 95%CI:3.29,22.51), witnessing maternal abuse (RRR = 5.56; 95%CI:2.13,14.52), and household substance misuse (RRR = 3.21; 95%CI:1.38,7.47) at or before the age of 18 were associated with very early sexual initiation. As for context of initiation, age at sexual initiation was younger if the man at initiation was a non-partner (ß = -0.88; 95%CI:-1.36,-0.40), was ≥3 years older (ß = -1.30; 95%CI:-1.82,-0.77), had pressured or forced sexual intitiation (ß = -1.09; 95%CI:-1.58,-0.59), and was under the influence of drugs/alcohol (ß = -0.97; 95%CI:-1.62,-0.32). Contextual variables at first sex, including being pressured or forced, and the man being ≥3 years older fully mediated the association between ACEs and early sexual initiation.

**Conclusions:**

This study highlights the critical need to develop interventions that reduce the impact of ACEs on women’s health and delay age at sexual initiation. Health education efforts are needed for clinicians and parents to identify and prevent childhood abuse and to identify and report sexual coercion and abuse for girls and adolescents.

## Introduction

*Adverse childhood experiences (ACEs)* are a range of negative childhood exposures before the age of 18 years, including trauma, abuse, and household dysfunction [1]. ACEs have gained recent attention due to emerging evidence of their complex and long-lasting impacts on health. Children exposed to four or more ACEs have up to 12 times the odds of having negative health outcomes in adulthood than children without such exposures [2]. ACEs have been linked to a variety of health disparities (e.g., alcohol and drug misuse, mental health disorders, sexual and reproductive health) in adolescence and adulthood [1, 3–7]. Specific to sexual risk behaviors, ACEs are associated with more lifetime sexual partners (≥30), perceiving oneself to be at risk for HIV, and engaging in HIV sexual risk behaviors (e.g., past year STI, condomless anal sex, exchange sex for money or drugs) [8, 9]. Moreover, the effect of ACEs on long-term health outcomes has been found to be stronger and more persistent among women than men [8, 10]. Despite the influence that ACEs may have on sexual and reproductive health, little (if any) research has considered the effect of ACEs on sexual initiation among women in the US.

One study suggests a link between ACEs and early sexual initiation [10]. Early sexual initiation, defined as sexual initiation before the age of 15, is an established risk factor for sexually transmitted infections (STIs), including HIV, and teen pregnancy [11–15]. Several mechanisms predispose young women with early sexual initiation to poor health outcomes. In terms of physiology, young women have an immature cervix that biologically predisposes them to STIs, including HIV, if exposed [16–18]. In terms of behavioral responses, sexual initiation marks a critical step in reproductive development. Behaviors adopted at sexual initiation, such as consistent condom use and partner negotiation, have been found to shape and influence sexual behaviors in adolescence and adulthood [19]. Likewise, early sexual initiation has also been associated with unintended pregnancy [20] and sexual violence later in life [21, 22].

Black women in the US experience alarming health disparities with regard to childhood trauma, abuse, and HIV/STIs. Black women experience five times more childhood abuse [23], and eight times more HIV diagnoses, than white women [24]. Moreover, Black youth tend to initiate sexual activity at a younger age than white youth [25]. The US national average age of sexual initiation is at 17 years old [26], whereas 8% of Black youth and 2% of white youth report sexual initiation before the age of 13 [27]. Despite these statistics, most of the evidence base around early sexual initiation is in international contexts, with early sexual initiation typically defined as sexual initiation before the age of 15 [20, 28]. US national data reveal that individual ACEs are associated with younger age at sexual initiation and increased risk for early sexual initiation, with greater effect estimates found among women and minorities than heterosexual men [10]. However, the mechanisms linking childhood trauma and neglect to early sexual initiation are largely unexplored.

The current study examines the effect of ACEs on early sexual initiation (categorical variable) and age at sexual initiation (continuous variable) among a sample of Black women seeking services from public health STD clinics in Baltimore, Maryland, US between November 2015 and May 2018. We apply the life course perspective, a developmental theory, to offer insight into how childhood traumas and abuse can affect health across multiple life stages. Specifically, we utilize the concept of cumulative trauma–that individuals experience multiple forms of trauma and abuse along their life course (e.g., in childhood, adolescence, and adulthood)–to guide the analysis and interpretation of our findings. We hypothesize that women with greater exposure to ACEs will have a greater likelihood of reporting an early sexual initiation (and a younger age at sexual initiation). The life course perspective also systematically directs attention to the importance of social and physical context in presenting risk factors for health disparities [29]. The “linked lives” principle in the life course perspective posits that individuals in the same household, family, or social network influence each other’s risk exposures and behaviors [30]. Likewise, exposure to ACEs would correspond to heightened exposure to contextual social and physical factors that heighten risk for earlier sexual initiation, such as older men with histories of mental health problems, substance use disorders, and sexual abuse. In this regard, we hypothesize that ACEs will work to increase risk of early sexual initiation (and decrease age at sexual initiation) via high-risk contextual variables present during sexual initiation.

## Methods

Data for this analysis come from, The ESSENCE Project: Examining Stress, Sexual Experiences, and Neighborhood Correlates of HIV Risk among Black Women (NICHD R01HD077891), a retrospective study examining the relationships between the built and social environment, sexual assault, and HIV risk behaviors among Black women in Baltimore City, Maryland. A Federal Certificate of Confidentiality was obtained to protect the privacy of participants by prohibiting the disclosure of identifiable, sensitive research information from outside requests and subpoenas. The ethical review boards of Johns Hopkins University and the University of California San Diego approved of the study design and consent procedures.

### Procedure

Black women (n = 312) were recruited for The ESSENCE Project between November 2015 and May 2018 in the waiting room of two Baltimore City STD clinics. Interested participants provided written consent to research staff and completed a computerized screener and HIV test in a private clinic setting to assess eligibility. Once eligibility was determined, a second written informed consent was obtained from participants explaining study procedures, confidentiality, and risk and benefits of participation. After participants provided informed consent and provided HIPAA Privacy Rule Authorization for access to their STI (including HIV) test results, research staff screened participants for eligibility. Inclusion criteria were: biologically female, between age 18–44, self-identified as Black, tested HIV-negative by clinical staff at enrollment, reported having sex with a man in the past 6-months, and have had at least two sexual partners in the past year or a high HIV risk sexual partner (i.e., used injection/non-injection drugs, have sex with men, been to prison, concurrent sex partner, had an STD, or was HIV-positive). A small percentage (3.3%, n = 8) identified as other or mixed race including Black/African descent, whereas a total of 8.7% (n = 21) self-identified as African-Caribbean, African born, or mixed race (versus African-American). During eligibility screening, women self-reported their HIV status, which was later confirmed via biological test results that were conducted by the Baltimore City STD clinics. Only one participant who was eligible for our study tested HIV-positive during recruitment, upon which she was unenrolled from the study and the STD clinic followed their standard protocol of providing warm referrals to HIV treatment and care services. The survey (~60 minutes) was administered via Audio Computer-Assisted Self-Interview (ACASI). Participants were paid a total of $45 ($10 for screening and $35 for questionnaire completion).

### Measures

Early sexual initiation was defined as: sex for the first time before age 15 [10, 20, 28]. Age at vaginal sexual initiation was measured by the question, “How old were you the first time you had vaginal sex with a man?” Age at anal sexual initiation was measured by the question, “How old were you the first time you had anal sex with a man?” Participants responded with the age at which first vaginal or anal sex occurred. Age at vaginal/anal sexual initiation was analyzed as a continuous variable. We further categorized age at vaginal/anal sexual initiation (using the youngest age at either vaginal or anal sexual initiation) into the following: (a) very early sexual initiation occurring at age 11–12, (b) early sexual initiation occurring at age 13–14, and (c) not early sexual initiation occurring at age 15 or older [10]. In n = 15 cases, vaginal and anal sexual initiation occurred at the same age, and in n = 3 cases, anal sexual initiation preceded vaginal sexual initiation. Although all cases of early sexual initiation could be considered child sexual abuse, a few women reported first sex during pre-pubescent stages of development [age 5 (n = 1), 8 (n = 1), and 10 (n = 4)]. We excluded these women from our analysis for clarity, as puberty (onset occurs after age 10 in girls) is the distinguishing factor between child molestation and statutory rape in most US states [31, 32].

ACEs were measured using Felitti’s (1998) original and standard 8-item scale assessing exposure to emotional, physical, and sexual abuse, and household dysfunction before age 18 (i.e., witnessing maternal abuse, household drug/alcohol misuse, household mental health problems, parental separation/divorce/loss, incarcerated household member) [1]. The sexual abuse ACE, was assessed with the question, “Did an adult or person at least 5 years older than you ever touch or fondle you, have you touch their body in a sexual way, or attempt or actually have oral, anal, or vaginal intercourse with you?” Participants responded ‘yes’ (1) or ‘no’ (0) to each item. Scores were summed to yield a total ACE score (ranged from 0 to 8). Responses to individual items were also examined.

Six vaginal/anal sexual initiation contextual variables were also assessed. Relationship with male at sexual initiation was assessed with the question, “how would you describe this person?” Partner was coded as any of the following responses: spouse, steady or main partner, and casual dating partner, whereas non-partner as a friend, stranger, father, stepfather or mother’s boyfriend, other male family member, or family friend or neighbor. Partner age difference was calculated by subtracting the male partner’s age at sexual initiation from the woman’s age at sexual initiation, and categorizing age difference based on the sample average difference in age. Women were, on average, 2.72 years younger than their main partner at sexual initiation (median of two years younger), therefore the cut-off was defined as the male partner being ≥three years older than the woman at the time of sexual initiation. Pressured or forced sexual initiation was assessed with the question, “how would you describe your first vaginal/anal sex experience?” Responses were dichotomized to measure pressured and/or forced sexual initiation. The response “wanted and not forced” was categorized into “no”, signifying that vaginal/anal sexual initiation was neither pressured nor forced. The following responses were categorized into “yes”, signifying that vaginal/anal sexual initiation was either pressured or forced: wanted but pressured, unwanted and pressured, unwanted and threatened with violence, unwanted and physically forced (hit, held down, slapped), and unwanted and forced to drink alcohol or take drugs. Women also reported yes or no to whether: (a) they (or the (b) male partner) used alcohol or drugs immediately before the first vaginal/anal sexual encounter.

### Analysis

**Fig 1:**
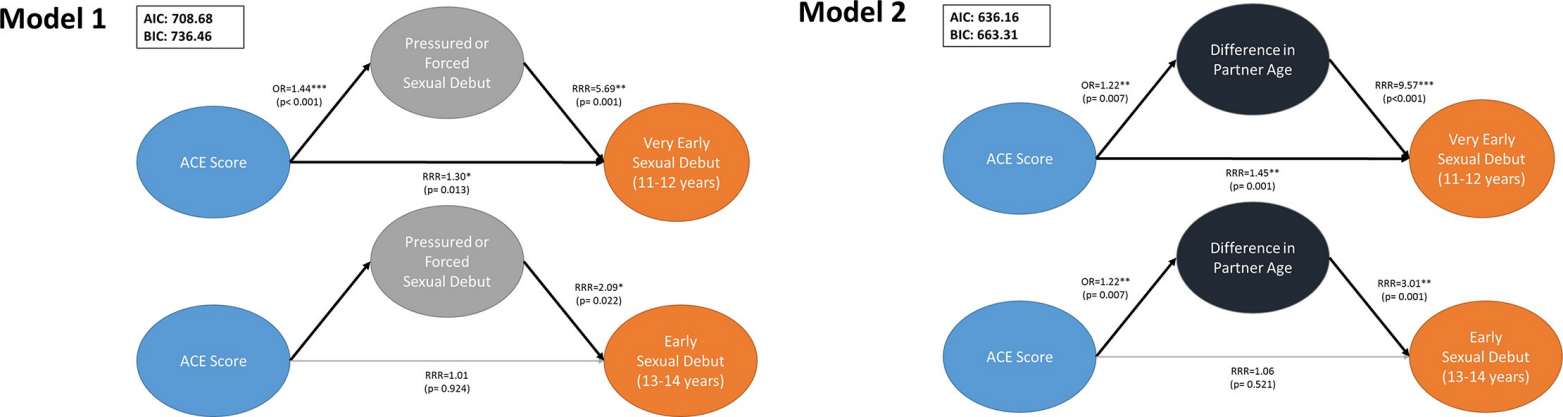
Pathway of mediation models of adverse childhood experience (ACE) cumulative score and age at sexual debut.

**Fig 2:**
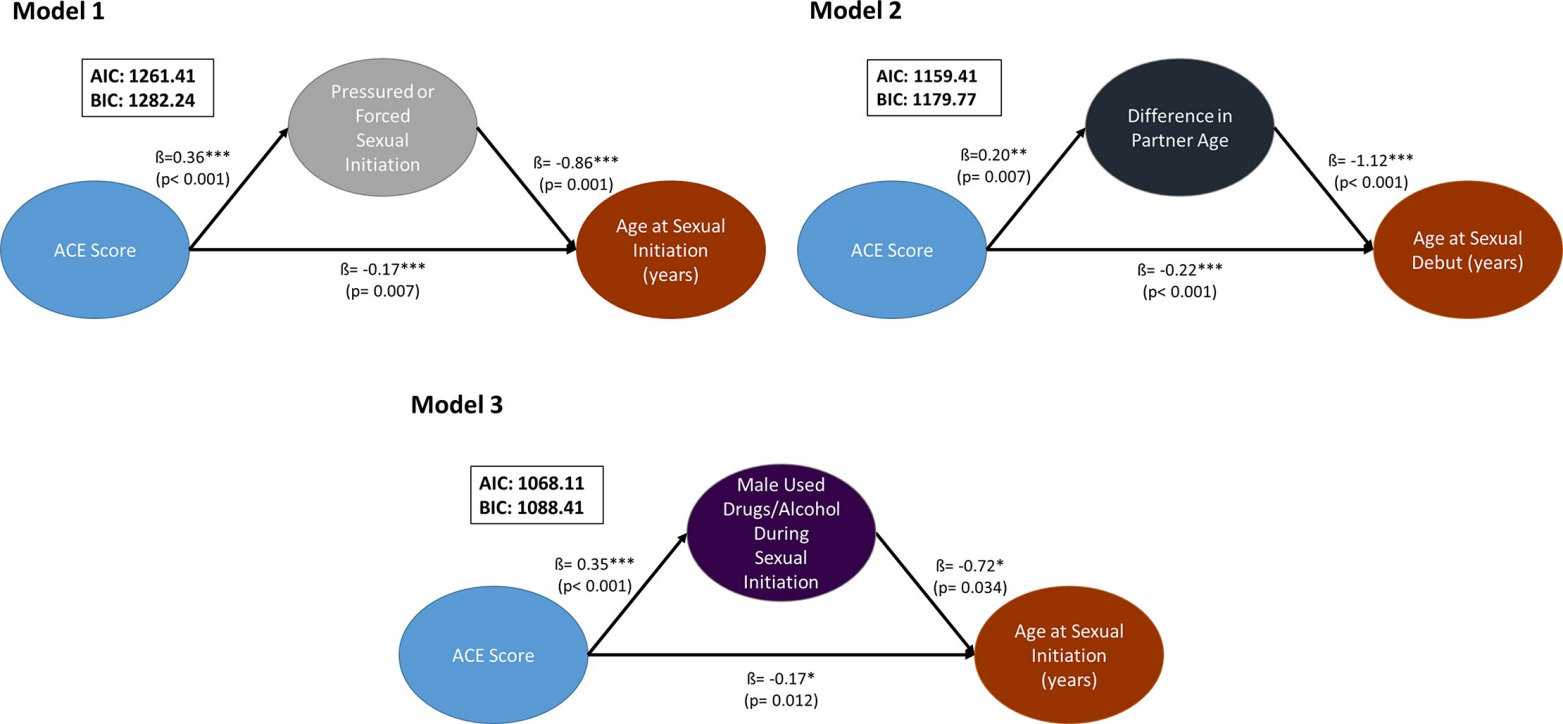
Pathway of mediation models of adverse childhood experience (ACE) cumulative score and age at sexual initiation.

All statistical analyses were run using Stata, version 15 [33]. Listwise deletion was performed to include individuals with complete data bringing the final analytic sample to n = 241 women with complete data. Upon further investigation, women included in the analysis were similar to women excluded from the analysis on most socio-demographic and sexual initiation characteristics, but were slightly more educated (85% had at least H.S. education vs. 73%), older at sexual initiation (14.9 vs. 13.6 years), and initiated sex with a younger male partner (age 17.5 vs. 20.4 years). First, we began our analysis with univariate and bivariate descriptive statistics (Table 1). Second, we estimated multinomial logistic and linear regression models to examine the association between ACEs (individual and sum ACE score) on early sexual initiation and age at sexual initiation (Table 2), respectively. Third, we assessed sexual initiation contextual variables as potential mediators of the association between ACE score and the two outcomes (early sexual initiation and age at sexual initiation). This included confirming that: 1) ACE score was significantly associated with the outcome variable (early sexual initiation OR age at sexual initiation), 2) ACE score was significantly associated with the mediator variable (we tested each of the six contextual variables), and 3) the mediator is significantly associated with the outcome with ACE score in the model [34–36]. Based on these preliminary steps, we identified the following contextual variables as potential mediators of the association between ACE score and sexual initiation: 1) pressured or forced sex, 2) difference in partner age, and 3) male used drugs/alcohol during sexual initiation (only for outcome of age at sexual initiation). Fourth, and lastly, we estimated mediation models using generalized structural equation modeling -gsem- command in Stata 15 to estimate path coefficients for multinomial (Fig 1) and binomial logistic outcomes (Fig 2).

**Table 1:** Socio-demographic characteristics and adverse childhood experiences (ACEs) of in Black women, Baltimore city Maryland (n = 241).

Variables		Very Early Sexual Initiation		Early Sexual Initiation		Not Early Sexual Initiation		Total Sample	
		11–12 years		13–14 years		15 years or older			
		(n = 28) 12%		(n = 70) 29%		(n = 143) 59%		(n = 241) 100%	
		n	%	n	%	n	%	n	%
***SOCIO-DEMOGRAPHICS***									
Age [mean(std dev.)]*		28.68	(1.25)	26.80	(0.82)	25.27	(0.50)	26.11	(6.39)
18–24		8	29	31	44	74	52	113	47
25–34		13	46	29	41	57	40	99	41
35–44		7	25	10	14	12	8	29	12
At least a high school education		23	82	60	86	121	85	204	85
Formally employed		13	46	35	50	92	64	140	58
Unstable housing*		4	14	9	13	5	4	18	8
Number of children									
0		13	46	36	51	72	50	121	50
1 to 2		9	32	26	37	54	38	89	37
3 or more		6	21	8	11	17	12	31	13
Individual income									
<$10,000		18	64	43	61	86	60	147	61
$10,000–29,999		9	32	23	33	43	30	75	31
>$30,000		1	4	4	6	14	10	19	8
***SEXUAL INITIATION CONTEXT***									
First sex with non-partner***		22	81	32	46	57	40	111	46
Partner ≥ 3 years in age***		17	74	27	44	27	20	71	32
First sex pressured/forced***		20	74	30	43	37	26	87	37
Partner used alcohol/drugs*		8	35	11	17	18	14	37	17
Woman used alcohol/drugs		5	19	8	12	12	8	25	11
***ACE CATEGORIES***									
Emotional abuse*		12	43	14	20	25	17	51	21
Physical abuse**		12	60	3	10	11	24	26	27
Sexual abuse		12	60	14	45	12	26	38	39
Witness maternal abuse**		10	36	9	13	13	9	32	13
Household substance misuse		13	46	20	29	30	21	63	26
Household mental health problems*		9	32	14	20	26	18	49	20
Parental separation or divorced		13	46	28	40	51	36	92	38
Incarcerated household member		9	32	13	19	35	24	57	24
ACE Score**^a^		3.21	(0.49)	1.64	(0.22)	1.42	(0.14)	1.69	1.95
0		7	25	27	39	59	41	93	39
1		3	11	11	16	34	24	48	20
2		2	7	15	21	17	12	34	14
3		2	7	7	10	15	10	24	10
4		2	7	3	4	8	6	13	5
5		6	21	3	4	5	4	14	6
6		4	14	3	4	3	2	10	4
7		1	4	0	0	1	1	2	1
8		1	4	1	1	1	1	3	1

Source: The ESSENCE Project 2015–2017; a p-value for statistical difference between very early/early/not early sexual initiation using X^2^ for comparisons among categorical variables (unless there is a zero cell) & the F-statistic for comparison of means among continuous variables

*p-value≤0.05

**p-value≤0.01

***p-value≤0.001

mean(standard deviation) reported for continuous variables; columns may add to more than 100% due to rounding

**Table 2:** Multinomial logistic and linear regression of early sexual initiation on ACEs among Black women, Baltimore city Maryland (n = 241).

Variables	Very Early Sexual Initiation		Early Sexual Initiation		Age at Sexual Initiation	
	11–12 years		13–14 years		(Continuous)	
	RRR	95% CI	RRR	95% CI	Coeff.	95% CI
**ACE Score (Continuous)**	1.49***	(1.23, 1.80)	1.07	(0.92, 1.25)	-0.26***	(-0.38, -0.13)
***ACEs (before age 18 years)***						
Emotional abuse	3.71**	(1.55, 8.89)	1.16	(0.56, 2.40)	-0.85**	(-1.44, -0.26)
Physical abuse	9.45***	(3.56, 25.12)	0.54	(0.14, 1.99)	-1.67***	(-2.43, -0.90)
Sexual abuse	8.60***	(3.29, 22.51)	2.79*	(1.21, 6.42)	-1.62***	(-2.27, -0.98)
Witness maternal abuse	5.56***	(2.13, 14.52)	1.48	(0.60, 3.64)	-1.16**	(-1.87, -0.45)
Household substance misuse	3.21**	(1.38, 7.47)	1.51	(0.78, 2.92)	-0.81**	(-1.36, -0.26)
Household mental health problems	2.25	(0.91, 5.57)	1.13	(0.55, 2.32)	-0.50	(-1.11, 0.11)
Parental separation or divorced	1.56	(0.69, 3.54)	1.23	(0.69, 2.22)	-0.33	(-0.84, 0.18)
Incarcerated household member	1.45	(0.60, 3.49)	0.70	(0.34, 1.42)	-0.09	(-0.67, 0.49)
***Sexual Initiation Context***						
First sex with non-partner	6.64***	(2.38, 18.54)	1.27	(0.71, 2.26)	-0.88***	(-1.36, -0.40)
Partner ≥3 years in age	11.33***	(4.08, 31.49)	3.09**	(1.60, 5.94)	-1.30***	(-1.82, -0.77)
First sex pressured/forced	8.03***	(3.14, 20.54)	2.11*	(1.15, 3.86)	-1.09***	(-1.58, -0.59)
Partner used alcohol/drugs	3.38*	(1.25, 9.11)	1.34	(0.59, 3.04)	-0.97**	(-1.62, -0.32)
Woman used alcohol/drugs	2.46	(0.79, 7.67)	1.42	(0.55, 3.65)	-0.64	(-1.44, 0.17)

Source: The ESSENCE Project 2015–2017; Comparison category for multinomial logistic regression is sexual initiation at 15 years of age or older; ACEs = Adverse Childhood Experiences; RRR = relative risk ratio; 95% CI = 95% Confidence Interval

*p-value≤0.05

**p-value≤0.01

***p-value≤0.001

## Results

Table 1 reports the socio-demographic, sexual initiation, and ACE characteristics of our sample. Forty-one percent of our sample experienced early sexual initiation (12%-very early sexual initiation; 29%-early sexual initiation). Women who experienced early or very early sexual initiation were significantly more likely to be unstably housed, compared to women who did not experience early sexual initiation. The contextual characteristics of sexual initiation indicate that most women who experience very early sexual initiation do so because they are pressured or forced (74%), highlighting the critical role of sexual violence as the introduction of their sexual relations. These pressured and forced first sexual encounters were mainly with non-partners (81%), who were at least three years older than them (74%), and among more than a third used alcohol and/or drugs during the sexual encounter (35%).

In terms of ACEs, 61% of all women experienced at least one ACE. Specifically, among our total sample of women, 39% reported sexual abuse, 38% reported parental separation or divorce, 27% reported physical abuse, 26% reported household alcohol/drug misuse, 24% reported an incarcerated household member, 21% reported emotional abuse, 20% reported household mental illness, and 13% reported witnessing domestic violence during childhood/adolescence (≤18 years old). Women who experienced very early sexual initiation reported significantly more exposure to emotional abuse, physical abuse, witnessing maternal abuse, and household mental health problems than their counterparts. Women who experienced very early sexual initiation reported significantly more ACE exposure (mean = 3.21; std.dev. = 0.49) than women with early sexual initiation (mean = 1.64; std.dev. = 0.22) and women without early sexual initiation (mean = 1.42; std.dev. = 0.14).

Table 2 reports the relative risk ratios of multinomial logistic regression models that examine the association between ACEs (individual and cumulative ACE score) and early sexual initiation. In terms of cumulative ACE score, each additional ACE reported was associated with a greater relative risk of experiencing very early sexual initiation for women (RRR = 1.49; 95%CI: 1.23,1.80). Cumulative ACE score was not significantly associated with early sexual initiation versus not early sexual initiation, but the association trended in the same direction. Taking a closer look at the bivariate association between individual ACEs and early sexual initiation, women who reported emotional abuse (RRR = 3.71; 95%CI: 1.55,8.89), physical abuse (RRR = 9.45; 95%CI: 3.56,25.12), sexual abuse (RRR = 8.60; 95%CI: 3.29,22.51), witnessing maternal abuse (RRR = 5.56; 95%CI:2.13,14.52), and having household substance misuse (RRR = 3.21; 95%CI: 1.38,7.47) at or before the age of 18 had a greater relative risk ratio of *very* early sexual initiation (versus not early sexual initiation), compared to women not reporting each ACE. Women who reported sexual abuse (RRR = 2.79; 95%CI: 1.21,6.42) at or before the age of 18 had a greater relative risk ratio of early sexual initiation (versus not early sexual initiation), compared to not reporting sexual abuse as an ACE.

We then estimated bivariate linear regression models to examine the association between ACEs (individual and cummulative ACE score) and age at sexual initiation (as a continuous outcome; Table 2). In terms of cumulative ACE score, each additional ACE reported was associated with a decrease in age at sexual initiation by about three months (β = -0.26; 95%CI: -0.38,-0.13). All of the individual ACE items were associated with younger age at sexual initiation. Specifically, in order of effect size, experiencing physical abuse (β = -1.67; 95%CI: -2.43,-0.90), sexual abuse (β = -1.62; 95%CI: -2.27,-0.98), witnessing maternal abuse (β = -1.16; 95%CI: -1.87,-0.45), emotional abuse (β = -0.85; 95%CI: -1.44,-0.26), and household substance misuse (β = -0.81; 95%CI: -1.36,-0.26) were associated with earlier sexual initiation.

We then estimated the bivariate associations between sexual initiation contextual variables and early sexual initiation (Table 2). In terms of very early sexual initiation, having a male partner that was ≥3 years older (RRR = 11.33; 95%CI: 4.08,31.49), having a pressured or forced sexual initiation (RRR = 8.03; 95%CI: 3.14,20.54), sexual initiation with a male non-partner (RRR = 6.64; 95%CI: 2.38,18.54), and having a male partner using drugs or alcohol during sexual initiation (RRR = 3.38; 95% CI:1.25,9.11) were associated with a greater relative risk ratio of experiencing very early sexual initiation. In terms of early sexual initiation, having a male partner that was ≥3 years older (RRR = 3.09; 95%CI: 1.60,5.94) and having a pressured or forced sexual initiation (RRR = 2.11; 95%CI: 1.15,3.86) were associated with a greater relative risk ratio of experiencing early sexual initiation.

We also estimated linear regression models to understand how sexual initiation contextual variables affected age at sexual initiation (Table 2). Having a male partner that was ≥3 years older (β = -1.30; 95%CI: -1.82,-0.77), having a pressured or forced sexual initiation (β = -1.09; 95%CI: -1.58,-0.59), having a male partner that used drugs or alcohol during sexual initiation (β = -0.97; 95%CI: -1.62,-0.32), and sexual initiation sexual initiation with a male non-partner (β = -0.88; 95%CI: -1.36,-0.40) were significantly associated with younger age at initiation.

Fig 1 illustrates three mediation models between ACE cumulative score, sexual initiation contextual variables (pressured or forced sexual initiation and difference in partner age) and early sexual initiation (categorical variable). In all three models the contextual variables fully mediated the association between ACE score and early sexual initiation, but not very early sexual initiation. In Model I (pressured or forced sexual initiation) and Model II (difference in partner age), contextual variables partially mediated the association between ACE score and very early sexual initiation; the association was attenuated in magnitude and significance.

Fig 2 illustrates four mediation models between ACE cumulative score, sexual initiation contextual variables (pressured or forced sexual initiation, difference in partner age, and male used drugs/alcohol during sexual initiation), and age at sexual initiation (continuous variable). The association between ACE cumulative score and age at sexual initiation remained significant in all four models, but with an attenuated effect size. ACE cumulative score was associated with significantly greater odds of the man being ≥3 years older, pressuring or forcing the sexual initiation, and using drugs or alcohol during sexual initiation. Contextual variables were associated with a nine to 16-month decrease in age at sexual initiation and remained significant mediators in the model. ACE score remained significantly associated with age at sexual initiation, but the association was slightly attenuated all models compared to the bivariate model.

## Discussion

This study analyzes the association between adverse childhood experiences (ACEs) and early sexual initiation, with three key findings. First, we find a critically high prevalence of early and forced sexual initiation among our sample of Black women in Baltimore, MD compared to other US-based studies. Second, our study describes exposure to ACEs among our sample and finds that ACEs are significantly associated with an increase in likelihood of very early sexual initiation and decreased age at sexual initiation. Third, we identified contextual factors at sexual initiation that potentially mediate the association between ACEs and very early sexual initiation and age at sexual initiation. We situate our findings within the context of health disparities and public health interventions.

We find a high prevalence of early sexual initiation among our sample of Black women in Baltimore, MD (41% - 12% very early sexual initiation, 29% early sexual initiation). Although we analyze data from a small, retrospective sample of Black women, these statistics tower over those reported in a nationally representative sample of men and women (10% - 3% very early sexual initiation and 7% early sexual initiation) [10]. Our study identifies forced or pressured sex as a key factor present during first sexual intercourse among women who report early sexual initiation. The prevalence of forced or pressured sexual initiation was much greater among women with very early (74%) and early sexual initiation (43%) compared to findings from a global literature review (13% coerced sexual initiation in the US and 5–46% in low- and middle-income countries) [37]. These disparities have critical implications for sexual and reproductive health of young Black women in Baltimore. Early sexual initiation and coerced sexual initiation confer great risk for HIV/STI infection and sexual risk behaviors (i.e., having a risky sexual partner in the past five years, having sexual intercourse under the influence of drugs and alcohol, having multiple sexual partners in the past year) [21, 38]. Despite having a very high rate of HIV among Black women in Baltimore, the association between ACEs and sexual risk of HIV has not been previously explored.

To our knowledge, this is the first study to contrast early sexual initiation and very early sexual initiation with respect to exposure to ACEs among a sample of Black women in the US. Our exploratory study provides support for the hypothesis that women who report greater exposure to ACEs in childhood and adolescence may have a greater risk of experiencing very early sexual initiation (11–12 years old), but not early sexual initiation (13–14 years old). Cumulative ACE score was also associated with a significantly younger age at sexual initiation, increasing women’s length of potential exposure to HIV/STIs [39, 40]. Although these findings are consistent with the limited research linking ACEs to early sexual initiation [8, 10]–it is noteworthy that ACE score was not significantly associated with sexual initiation at 13–14 years old in our study. We found that the ACEs with strongest association with very early sexual initiation (and younger age at sexual initiation) included experiencing abuse (physical, sexual, or emotional), witnessing maternal abuse, and living in a household where someone misused substances, supporting previous research [41, 42]. Furthermore, we acknowledge the complexity and overlap of individual ACEs, and that girls are particularly vulnerable to more severe and long-lasting health outcomes as a result of exposure to ACEs compared to boys [10, 43].

Despite the overall trend in significant associations between ACEs and early sexual initiation, little is known about the mechanisms by which ACEs work to increase sexual risk for early sexual initiation. Our study identified pressured or forced sex was a pervasive factor in first sexual relations among our sample of Black women, highlighting the need to address sexual violence among minors. Other contextual factors associated with very early sexual initiation involved male characteristics. Specifically, women were at greater relative risk of very early sexual initiation if it was with a man who was ≥3 years older than her, if he was a non-partner, and if he used drugs or alcohol at the time of sexual initiation. Similar contextual sexual risk behaviors were associated with an overall younger age at sexual initiation.

Our mediation analysis revealed that the association between ACE score and early sexual initiation can be explained by contextual factors present at first sex. Namely, greater exposure to ACEs in childhood and adolescence increased the likelihood of that early sexual initiation occurred due to pressured or forced sex, and with a man who was ≥3 years older. These factors also partially mediated the association between ACE score and age at sexual initiation, along with the man using drugs or alcohol at the time of sexual initiation. Noteworthy, is that the effect size of the contextual characteristics on age at sexual initiation were much greater in magnitude than that of ACE score. These findings support hypotheses posited by the life course perspective, as exposure to ACEs confers risky social and physical contexts that place women at greater vulnerability of experiencing early sexual initiation [29]. The salience of male sex partner characteristics in mediating the association between ACE score and early sexual debut supports the “linked lives” life course perspective principle, where individuals in the same family or social network influence risky contexts and age at sexual initiation [30].

Our results point to the importance of interrupting the cycle of abuse women experience throughout their life course. According to the concept of cumulative trauma, women who report childhood abuse, are at greater risk of also experiencing pressured or forced early sexual initiation, and other forms of sexual violence as adolescents and adults. Cumulative trauma is thought to increase women’s likelihood of becoming infected with STIs/HIV, being diagnosed with mental health disorders (post-traumatic stress disorder, depression, anxiety), and developing substance use disorders [21]. The American Academy of Pediatrics recommends screening adolescents for ACEs in primary care settings, a policy recommendation supported by this study [44]—our study suggests starting this screening well before the age of 11–12 to identify abuse early on. Once a history of ACEs is determined, adolescents can be offered interventions for trauma, as well as anticipatory guidance around sexual health. It is also critical that clinicians and parents be vigilant for and prevent risk factors for childhood abuse. Additionally, girls and their parents need to be educated around sexual coercion, sexual abuse, and provided the tools to delay sexual initiation, as girls and women often do not recognize sexual abuse. For example (data not reported), only around half (n = 14) of the n = 25 women who experienced (very) early sexual initiation with a man at least 5 years older than them identified this as sexual abuse in the ACEs scale.

Our findings should be taken in light of several critical limitations. First, because of the retrospective and cross-sectional design of our study, the directionality of associations cannot neatly be determined. For example, ACEs were measured up to age 18, whereas early sexual initiation was defined as having first sex between the age of 13 and 14. We assume that ACEs are likely present throughout the individual’s life, but it is possible that, in some cases, sexual initiation preceded the experience of certain ACEs. Although this is a serious limitation, it further justifies the need for longitudinal research that is initiated during adolescence, to better understand the associations between ACEs and sexual and reproductive health outcomes. Despite this limitation, our findings using the continuous ACE score held up in magnitude and significance even when the sexual abuse ACE was omitted from consideration in sensitivity analyses. Moreover, our data offer rich measures of sexual initiation context that are not considered in most longitudinal and cohort studies. Second, the results may not be generalizable to women in the United States as the study included a limited sample of 241 HIV-negative, Black women who reported sexual risk for HIV and were recruited from public STI clinics in Baltimore. Lastly, a limitation was that individual ACE measures captured a wide scope. For example, the sexual abuse ACE question included touching, fondling, and oral, anal, and vaginal sex with a man at least 5 years older; this measure encompasses experiences that could be of very different natures. The ACE scale could perhaps include early sexual initiation as a new ACE variable, as it will bring attention to the fact that, even when it occurs among peers, early sexual initiation may significantly affect health later in adulthood.

In summary, our study highlights the importance of understanding the context and childhood correlates of early sexual initiation in vulnerable populations of girls and young women. There is an urgent need to develop interventions to mitigate the impact of ACEs on adolescent and women’s sexual and reproductive health, especially in delaying sexual initiation among Black women. The incorporation of ACE screening during routine care can help identify the most at-risk individuals to facilitate referrals, undergo targeted interventions, and help to delay sexual initiation and its associated consequences [45, 46]. Parent-based interventions and adolescent-only interventions have demonstrated success in delaying sexual initiation. Of parent-based interventions, the most effective components in reducing sexual risk behavior and delaying sexual focus on improving effective communication and supportive parenting strategies [47, 48], specifically improving mother-child connectedness and making young women feel that adults or parents cared [49, 50]. Of the adolescent-only interventions, the most effective components in delaying sexual initiation involved comprehensive sex education as well as communication skills training (i.e., sexual communication assertiveness and negotiation skills) [47, 51]. Furthermore, there is a critical need for longitudinal research initiated during early adolescence to better unpack the causal mechanisms that connect ACEs to early sexual initiation.
